# Draft Genome Sequence of a Human-Associated Isolate of *Methanobrevibacter arboriphilicus*, the Lowest-G+C-Content Archaeon

**DOI:** 10.1128/genomeA.01181-13

**Published:** 2014-01-23

**Authors:** Saber Khelaifia, Marc Garibal, Catherine Robert, Didier Raoult, Michel Drancourt

**Affiliations:** Aix Marseille Université, URMITE, UMR63 CNRS 7278, IRD 198, INSERM 1095, Marseille, France

## Abstract

We report the draft genome sequence of *Methanobrevibacter arboriphilicus* strain ANOR1, isolated from the human gut. Its 2.21-Mb genome exhibits a 25.46% G+C content, the lowest value among archaea. The genome of *M. arboriphilicus* contains a total of 2,111 open reading frames and three clusters of regularly interspaced short palindromic repeat (CRISPR) loci with associated Cas proteins.

## GENOME ANNOUNCEMENT

*Methanobrevibacter arboriphilicus* was initially isolated from wet wood of living trees (1) and from paddy field soil (2). *M. arboriphilicus* DNA has been detected in colonic mucosa collected in human patients, but no isolate was made from these specimens (3). Recently, we isolated *M. arboriphilicus* strain ANOR1 from the human gut (4), using strict anaerobic incubation at 37°C (pH 7.5) and 1.5 g/liter NaCl. Sequencing the 16S ribosomal DNA (GenBank accession no. KC616344) yielded 99% sequence similarity with the reference strain *M. arboriphilicus* DSM 1125 (GenBank accession no. AY196665). Strain ANOR1 therefore represents the first *M. arboriphilicus* isolate from the human gut.

The complete genome of *M. arboriphilicus* was sequenced by combining shotgun and 3-kb paired-end libraries using high-throughput 454 pyrosequencing (454 Life Sciences-Roche, Boulogne-Billancourt, France). The sequence reads were assembled using the Newbler assembler 2.8 (20120726_1306) (Roche); 24 contigs were generated into four scaffolds, and gaps were closed by PCR on genomic DNA. Preliminary open reading frame (ORF) prediction was conducted by automated annotation with Glimmer (http://www.cbcb.umd.edu/software/glimmer/) and RAST (5). The annotation was manually curated using BLAST and the NCBI NR database. The clusters of regularly interspaced short palindromic repeat (CRISPR) finder (http://crispr.u-psud.fr/Server/) was used to detect and identify CRISPR repeat and spacer sequences in the genome. The histogram of ORF lengths was realized using the MS Office Excel program.

The *M. arboriphilicus* genome consists of one circular 2,216,660-bp chromosome with no evidence of extrachromosomal DNA, as confirmed by pulsed-field gel electrophoresis and PCR sequencing embracing the contigs. A total of 2,111 ORFs were found, most of them presumably encoding proteins involved in DNA/RNA metabolism, synthesis and degradation of proteins, biosynthesis of nucleotides, amino acids, fatty acids, vitamins, and cofactors, and energy metabolism. One 3-kb and one 9-kb ORF yielded BLAST similarities of 30% to 69% with a sequence of *Methanobacterium ruminantium*, with coverages of 40% to 98%. *M. arboriphilicus* also encodes nonribosomal synthesis proteins, as well as bacteriocins. *M. arboriphilicus* has the capacity to inhibit other gut microbiotal inhabitants. Three CRISPR loci and the associated proteins (Cas) might confer resistance against the intrusion of mobile elements, such as viruses and plasmids (1, 6).

Of note, *M. arboriphilicus* ANOR1 exhibits a 25.46% G+C content, the lowest of the sequenced archaeal genomes. As an example, other human-associated archaea exhibit higher C+G contents, including *Methanomassiliicoccus luminyensis* (60.5%) (7), *Methanobrevibacter millerae* (26 to 38%) (8), *Methanobrevibacter smithii* (31%) (6), *Methanosphaera stadtmanae* (28%) (9), and *Methanobrevibacter oralis* (28%) (10). Among archaea at large, the higher G+C value of 66% is observed in *Halobacterium salinarum* (11). The G+C content of nucleic acids was shown to correlate with the stability of their double-helix (12). In particular, microorganisms with a high G+C content have been demonstrated to be more resistant to heat (12). However, that may not be the ultimate force regarding G+C content in archaea, as *Pyrococcus furiosus*, an archaeon with 38% G+C content, lives at 97°C (13).

### Nucleotide sequence accession numbers.

The *M. arboriphilicus* ANOR1 (CSUR P1715) genome sequence has been deposited in EMBL under the accession no. CBVX010000001 to CBVX010000005. The whole-genome shotgun project has been deposited in GenBank under the accession no. CBVX000000000.
